# Increasing fibre in white flour and bread: Implications for health and processing

**DOI:** 10.1111/nbu.12648

**Published:** 2023-10-31

**Authors:** Peter R. Shewry, Edward J. M. Joy, Lucia Segovia De La Revilla, Annalene Hansen, Joe Brennan, Alison Lovegrove

**Affiliations:** ^1^ Rothamsted Research Harpenden UK; ^2^ London School of Hygiene & Tropical Medicine London UK; ^3^ Aberystwyth University, Penglais Aberystwyth UK; ^4^ UK Flour Millers London UK

**Keywords:** dietary fibre, health benefits, wheat, white bread, wholegrain

## Abstract

Dietary fibre is beneficial for human health, but dietary intakes are below recommended levels in most countries. Cereals are the major source of dietary fibre in Western diets, with bread providing about 20% of the daily intake in the United Kingdom. Despite the promotion of fibre‐rich wholegrain products, white bread (which has a lower fibre content) remains dominant in many countries due to cultural preferences. Increasing the fibre content of white bread and other products made from white flour is therefore an attractive strategy for increasing fibre intake. This can be achieved by exploiting genetic variation in wheat without major effects on the processing quality or the consumer acceptability of products. Modelling data for food consumption in the United Kingdom shows that increasing the fibre content of white flour by 50% (from about 4% to 6% dry weight) and in wholegrain by 20% will increase total fibre intake by 1.04 g/day and 1.41 g/day in adult females and males, respectively. Furthermore, in vitro studies indicate that the increased fibre content of white bread should reduce the rate of starch digestion and glucose release in the human gastrointestinal tract.

## INTRODUCTION: WHEAT GRAIN AND FIBRE

The importance of dietary fibre for human health has become increasingly recognised over the past half century, with high fibre intake being associated with reduced risk of a range of chronic diseases, including cardiovascular disease, type 2 diabetes and several types of cancer. These benefits are summarised in excellent reviews and meta‐analyses (Gill et al., 2021; Veronese et al., 2018) and will not be discussed in detail here. A critical review of the evidence for the health benefits of carbohydrates, including dietary fibre, was prepared by the UK Scientific Advisory Committee on Nutrition (SACN) (2014). Nevertheless, most wheat‐consuming countries remain deficient in their intake of fibre. For example, UK adults consume about 20 g of fibre per day, compared to a recommended intake of 30 g per day.

Almost all the fibre in the human diet comes from plant‐based foods, with a small contribution from edible fungi. Furthermore, the most important source of fibre in most diets is cereals. For example, in the United Kingdom, cereals account for about 40% of the daily intake of fibre (Bates et al., 2010), with about half of this being provided by bread (Lockyer & Spiro, 2020).

Consequently, much of the research on the benefits of fibre has focused on cereals, particularly wholegrain wheat (Aune et al., 2016; Seal & Brownlee, 2015). This is because wholegrain wheat products contain much higher contents of dietary fibre than refined white bread, often over 15% dry weight, compared to about 4% dry weight in white bread.

The contribution of bread to fibre intake therefore varies between countries based not only on total bread consumption but also on the popularity of wholegrain products. For example, higher values than those for the United Kingdom have been reported for diets in Poland, where a higher proportion of wholegrain breads are consumed, with 48.5% of dietary fibre coming from cereals, including 35.4% from bread, rolls and bread products (Laskowski et al., 2019).

## INCREASING THE CONSUMPTION OF WHOLEGRAIN BREAD

The large volume of evidence for the role of cereal grain fibre in reducing the incidence of chronic diseases led to landmark approval of health claims for wholegrains by the US Food and Drink Federation (FDA) in 1999 (FDA, 1999), followed by the approval of several claims for cereal grain fibres by the European Food Safety Authority (EFSA) (https://ec.europa.eu/food‐feed‐portal/screen/health‐claims/eu‐register). These approvals stimulated the commercial development of wholegrain products and underpinned dietary recommendations by government agencies and/or regulatory bodies to consumers to increase the consumption of wholegrain foods. For example, the UK Eatwell Guide suggests that consumers should choose higher fibre or wholegrain varieties of starchy foods. Industry campaigns have also been carried out, such as ‘Fibre February’ organised by the UK Flour Millers, which emphasises the health benefits of fibre and encourages consumers to include wholemeal products, as well as other sources of fibre, into their diets.

Nevertheless, the strategy has failed to increase the proportion of wheat consumed as wholegrain products in the United Kingdom, with white bread remaining dominant and the consumption of wholemeal and brown breads actually declining.

**FIGURE 1:**
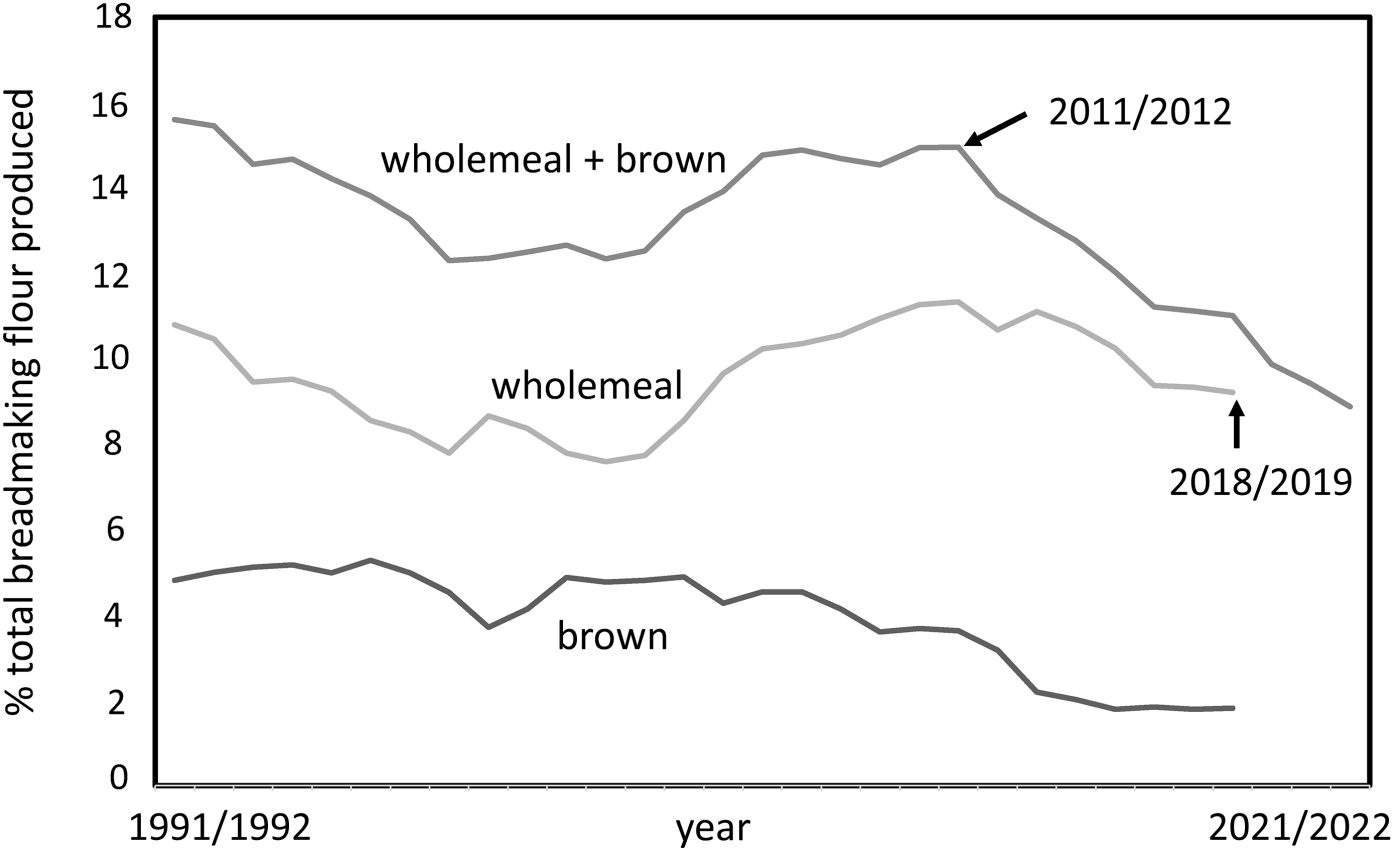
The production of wholemeal and brown breadmaking flours (tonnes) as a percentage of total breadmaking flours in the United Kingdom over the period 1991/1992–2022/2023. Separate data for brown and wholemeal flours are only available up to 2018/2019. ‘Brown’ flour and bread are not strictly defined but typically include some bran and germ corresponding to about 85% of the grain (compared to 78%–80% for white flour). Source: AHDB Cereal Usage Survey, ‘UK Flour Millers cereal usage’ dataset.

The total production of flour for breadmaking in the United Kingdom varies from year to year but averaged 2.75 million tonnes per year between 2017–2018 and 2021–2022. The proportion of wholemeal and brown breadmaking flours has decreased by about 40% since 2011/2012, from 14.9% of all breadmaking flours to 9.0% in 2022/2023, (Figure 1). Although the production of breadmaking flour has experienced a slight decline over this same period (from 2.93 million tonnes in 2011/2012), this does not account for this decrease. Similarly, although grain‐based foods account for over half of the dietary fibre intake in the United States, only 7% is from foods rich in wholegrain and fibre (Kranz et al., 2017).

This failure is not surprising, as other studies have shown that consumers are reluctant to change dietary habits that are often rooted in culinary and cultural traditions. For example, a study of consumers' perceptions and consumption patterns of salt in the United Kingdom concluded that ’consumption practices were largely driven by habit and lifestyle choices, rather than health considerations’ (Kenten et al., 2013). Although such considerations are of interest to sociologists and consumer scientists, the low uptake of wholegrain products despite vigorous promotion suggests that other strategies are required to increase fibre intake, the most logical and obvious being to improve the quality of widely consumed foods. In the case of wheat, this means increasing the fibre content of white bread. For this strategy to succeed and have a wide impact, there must be no effects on food cost, food quality, palatability and consumer acceptability (taste), and for the impact to be pro‐equity, it should reach disadvantaged groups and consumers whose current fibre intakes are low relative to others. We will therefore discuss whether this can be achieved.

## INCREASING FIBRE IN WHITE FLOUR

White flour is derived from the major storage tissue of the grain, called the starchy endosperm. This tissue accounts for about 80% of the grain and itself consists of about 80% starch, with about 10%–15% protein, about 4% dietary fibre, soluble sugars and minor components including minerals and vitamins (particularly B vitamins). The most widely accepted definition of dietary fibre is that proposed by the EU (Commission Directive 2008/100/EC, 28 October 2018): ’carbohydrate polymers with three or more monomeric units (to exclude mono‐ and disaccharides, simple sugars of one or two molecules) that are neither digested nor absorbed in the small intestine’, which ’may be closely associated in the plant with lignin or other non‐carbohydrate components’ which ‘when extracted with the carbohydrate polymers for analysis of fibre may be considered as fibre’. Hence, dietary fibre is a mixture of components that vary in their presence or absence, amounts and structures between different plant tissues and hence foods.

In fact, the fibre fraction in white flour is rather simple in composition compared with other foods. The major fibre components are cell wall polysaccharides, mainly arabinoxylan (pentosan), with smaller amounts of β‐glucan and cellulose, traces of other polysaccharides (xyloglucan, glucomannan and pectins) and no lignin (Table 1). Other fibre components in white flour are fructans (fructo‐oligosaccharides, mostly comprising 3–5 sugars) and arabinogalactan peptide (AGP) (which consists of a short peptide with three hydroxyproline residues that are *o*‐glycosylated with branched arabinogalactan chains).

**TABLE 1:** Approximate proportions of dietary fibre components in white flour.

	Amount and solubility	
	% dry weight	% soluble
Total dietary fibre	4	56–69^5^
Arabinoxylan^1^	1.9 (range 1.35–2.75)	25–50
β‐blucan^2^	0.2	30
Fructans^3^	1.5–1.7	100
Cellulose	<0.1	0
Arabinogalactan peptide (AGP)^4^	0.3	100

^1^ Gebruers et al. (2008).

^2^ Prins et al., 2023.

^3^ Haska et al. (2008) mean of analyses of three cultivars.

^4^ Calculated based on Loosveld et al. (1997).

^5^ Calculated based on 1.9% arabinoxylan of which between 25% and 50% is water‐soluble. The values do not include resistant starch, which accounts for about 1.2% of total starch (about 1% DM) in bread made from white flour.

**FIGURE 2:**
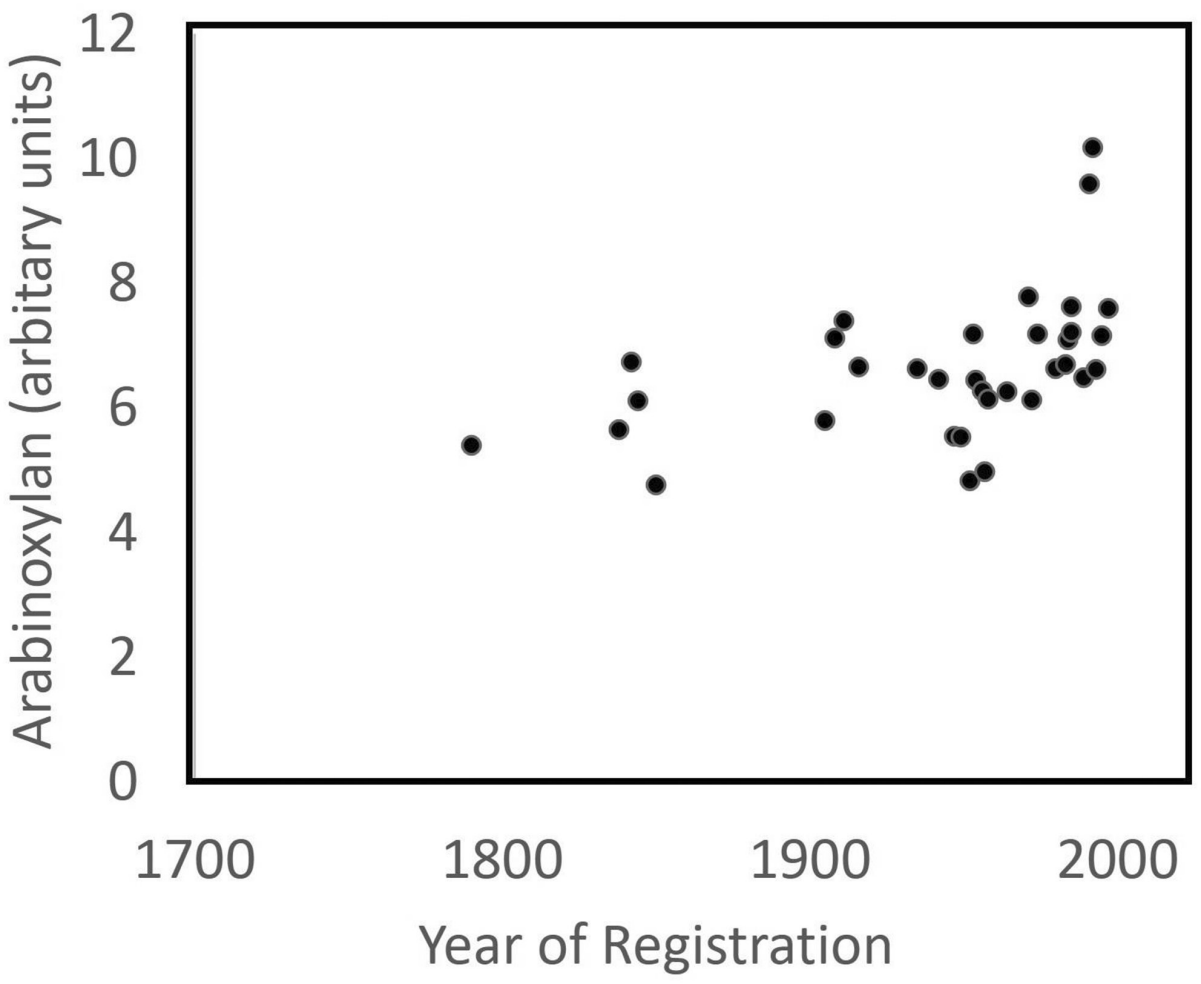
Mean content of arabinoxylan (% dry wt.) in white flours of wheat cultivars registered in the United Kingdom between 1790 and 2012 and grown in three replicated randomised field trials (2013–2014, 2014–2015, 2015–2016). Redrawn from Lovegrove et al. (2020) with permission.

Taken together, these components account for an average of about 4% of the dry weight of white flour, of which arabinoxylan is about half. Hence, arabinoxylan has been identified as a target for improvement. This strategy is based on two observations: the proportion of arabinoxylan in white flour varies about two‐fold between different varieties and samples (from about 1.35% to 2.7% dry weight) (Figure 2) and most of this variation (70%–80%) results from genetic differences between different types of wheat. The aim, therefore, is to increase the concentration of arabinoxylan in white flour from the current average of below 2% to about 4% dry weight, which would increase total fibre from about 4% to about 6% dry weight. In practice, this target has proved rather challenging because the genetic control of arabinoxylan in white flour is complex, with few major genes and confounding effects of the environment. Hence, it will be necessary to stack several genes to achieve the required levels of arabinoxylan.

However, it is clear that there is no relationship between the fibre content of the grain and the performance and yield of the crop, and hence no effects on production costs. In fact, comparisons of modern high‐yielding types of wheat with older traditional types, including ’ancient’ wheats (einkorn, emmer and spelt), have shown that modern wheats tend to have higher contents of arabinoxylan (Lovegrove et al., 2020; Shewry et al., 2020).

## EFFECTS OF INCREASED FIBRE ON PROCESSING

Arabinoxylan fibre has two physical properties which are relevant to processing. Firstly, about a quarter of the arabinoxylan present in white flour is water‐soluble and is the main contributor to the viscosity of water extracts of white flour (and dough). Secondly, it absorbs a high volume of water, about 10 times its own dry weight for the water‐insoluble fraction and 11 times its dry weight for the water‐soluble fraction. This affects the water absorption of flour, which is an important factor for bakers. The higher water absorption and dough viscosity are clearly of relevance to grain processing and are frequently raised when discussing strategies to increase the fibre content of white flour for UK bakers. However, in our experience, the variation in viscosity and water absorption associated with differences in the arabinoxylan content of white flours is within the range that UK millers and bakers currently observe between grain samples of breadmaking wheat.

Direct effects of arabinoxylan on breadmaking quality have been shown by comparisons of genotypes differing in the content of endogenous arabinoxylan (Hernandez‐Espinosa et al., 2020) and by experimental manipulation of arabinoxylan amount and properties (using supplementation and endoxylanase treatment) (reviewed by Courtin & Delcour, 2002). These studies show that high levels of water‐extractable arabinoxylan, especially large water‐extractable fragments, have positive effects on dough strength and loaf volume, while high levels of total arabinoxylan and water‐unextractable arabinoxylan may have negative effects.

Variation in the proportions of water‐extractable and water‐unextractable fractions in high arabinoxylan lines and the manipulation of these by endoxylanase treatment may therefore allow health benefits to be combined with improved breadmaking quality. However, it is crucial to demonstrate that these benefits can be delivered in high‐volume commercial breadmaking.

## CALCULATING THE IMPACT OF HIGHER FIBRE WHITE FLOUR ON FIBRE INTAKE AND HEALTH

Although the current recommended daily intake of dietary fibre in the United Kingdom is 30 g, the average daily consumption by UK adults is only about 20 g and white breads currently provide about 8%–10% of this (Roberts et al., 2018; Steer et al., 2008). The *National Diet and Nutrition Survey (NDNS)* generates information on food consumption and nutrient intakes among a nationally representative sample of the UK population, with *n* = 3558 participants in the most recently reported rounds 9–11 (Venables et al., 2022). Participants conduct an individual‐level, multi‐day recall of foods consumed, and this is matched to a national food composition dataset to generate data on nutrient intakes (Traka et al., 2020). Using these integrated datasets, it is possible to simulate the effects of increased fibre in wheat flour on dietary fibre intakes. Here, the total dietary fibre level used for white flour products was increased by 50% and for wholemeal products by 20%. The list of manipulated food items is provided in Table S1. The calculated data predict that the intake of fibre per day would increase by 1.41 g for males and by 1.04 g for females, with broadly similar proportional increases for different age groups (Table 2). We also calculate that this increase would enable an additional 4.3% of males (from 11.3% to 15.6%) to reach the intake target, but only an additional 1.7% of females (from 5.7% to 7.4%).

**TABLE 2:** Total dietary fibre intake and proportion of the population reaching the recommended fibre intake in the United Kingdom, calculated from National Diet and Nutrition Survey data (rounds 9–11).

		Fibre intake (g/day) mean (SE)		Population with fibre intake above recommended (%) mean (SE)	
		Current	Higher fibre	Current	Higher fibre
Gender	*n*				
Male	1636	19.7 (0.4)	21.1 (0.4)	11.3 (1.2)	15.6 (1.4)
Female	1922	17.2 (0.2)	18.2 (0.2)	5.7 (0.7)	7.4 (0.8)
Age					
Pre‐school (1.5–3 years)	306	10.4 (0.2)	11.1 (0.3)	11.7 (1.9)	14.2 (2.1)
Junior School (4–10 years)	725	14.4 (0.2)	15.4 (0.2)	13.7 (1.7)	19.6 (1.9)
Adolescents (11–18 years)	683	16.0 (0.3)	17.2 (0.3)	4.4 (1.1)	7.1 (1.3)
Adults (19–65 years)	1392	19.7 (0.3)	21.0 (0.3)	8.8 (1.0)	12.0 (1.1)
Older Adults (>65 years)	452	18.7 (0.4)	20.0 (0.4)	6.4 (1.4)	7.5 (1.5)

*Note*: The higher fibre scenario assumes an increase in the fibre content of wholemeal (+20%) and white (+50%) flours, with no changes in food item consumption.

Although these increases in fibre intake are modest compared to the increases in fibre intake required, they should be achievable without additional cost to farmers, processors and consumers. The question therefore is whether such modest increases would have any significant impact on health outcomes.

**FIGURE 3:**
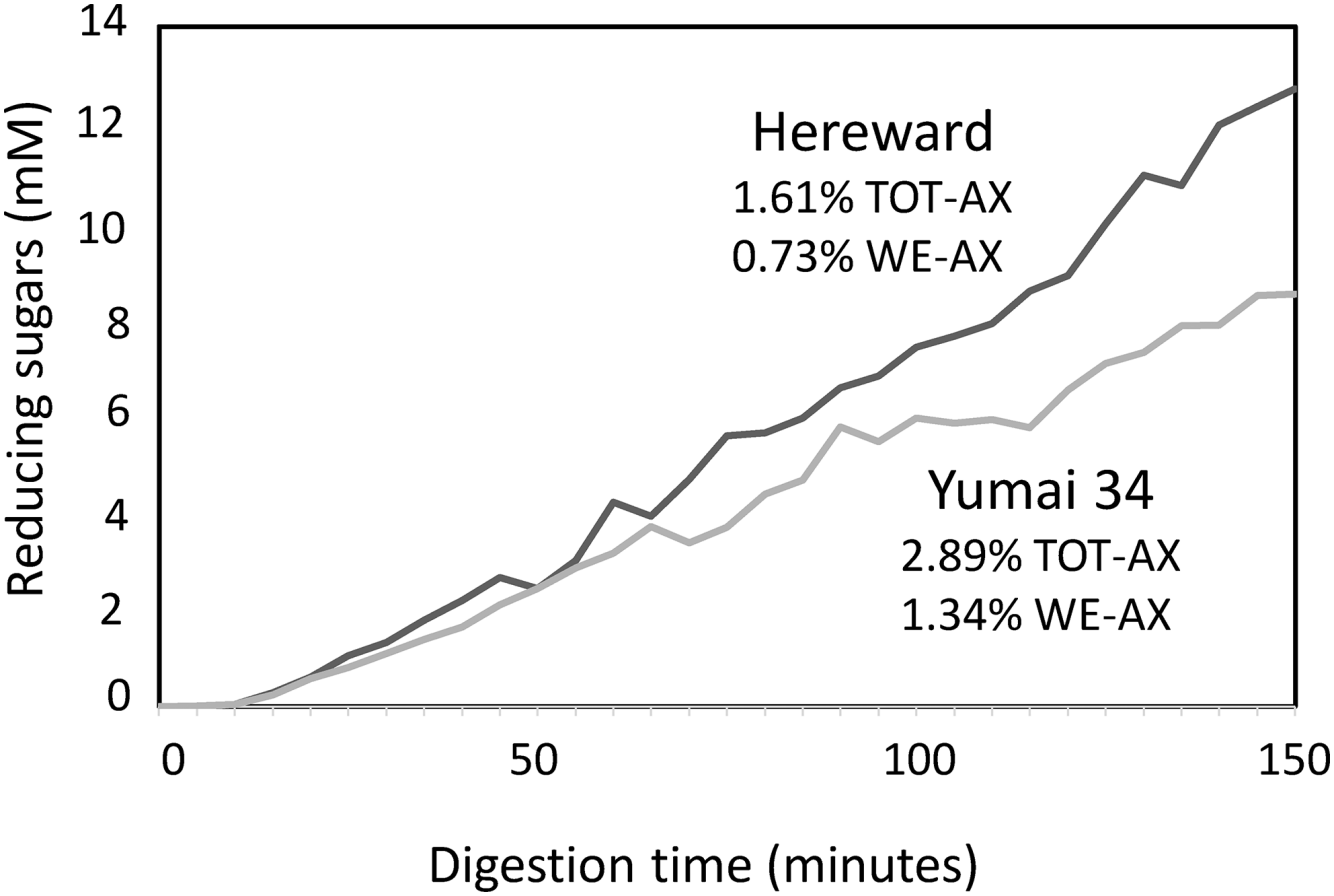
Simulated release of reducing sugars from bread made from white flours of the commercial UK cultivar Hereward and the high arabinoxylan Chinese cultivar Yumai 34 using a model duodenum system. TOT‐AX, total arabinoxylan; WE‐AX, water‐extractable arabinoxylan. Redrawn from Gouseti et al. (2019) with permission.

Evidence that such small differences may be relevant comes from in vitro digestion of breads made from the high arabinoxylan cultivar Yumai 34 (2.89% total and 1.34% water‐extractable) and the UK breadmaking cultivar Hereward (1.34% total and 0.73% water‐extractable arabinoxylan) (Figure 3) (Gouseti et al., 2019). Digestion in a model duodenal system after simulated gastric digestion showed a reduced rate of glucose release (from the digestion of starch) from bread made from Yumai 34 compared to the control Hereward bread. More recently, Ying et al. (2023) have shown significant effects of the addition of 2% (w/w) of arabinoxylan, 2% (w/w) of β‐glucan or 1% (w/w) of each of the two polymers on the rate of starch hydrolysis in bread.

The effect of arabinoxylan on the rate of starch digestion could result from reduced diffusion due to increased intestinal viscosity (resulting from the increased content of water‐extractable arabinoxylan) or from the effects of arabinoxylan on the food structure, resulting in a decreased rate of food breakdown and reduced accessibility of the substrate (starch) to digestive amylases. Irrespective of the mechanism, it suggests that relatively small increases in the content of arabinoxylan can have significant effects on glucose release from bread.

Despite the encouraging results discussed above, it is clear that further research is required to validate the approach and explore any potential beneficial effects on health. In particular, it is necessary to demonstrate that the increased fibre levels have effects in vivo, as opposed to in model systems, and to understand the mechanisms. Similarly, the arabinoxylan fibre present in white flour has a simple structure compared to arabinoxylan fractions prepared from bran, which have been more widely studied. It is therefore important to define the relationships between arabinoxylan structure and health benefits as well as quantify the effects of the total amount of arabinoxylan. These studies will require substantial funding and should be carried out in parallel with crop improvement if benefits are to be delivered in a timely fashion.

## CONSUMER ACCEPTABILITY OF HIGHER FIBRE WHITE BREAD

The ultimate factor determining the impact of ’higher fibre’ white bread on health is consumer acceptability. The dominant position of white bread in the United Kingdom demonstrates that conventional white bread has high consumer acceptability, and it is crucial for high uptake that increases in fibre should not have effects on this.

We are confident that the increases in fibre discussed above can be achieved without any effects on product quality (appearance, flavour, texture and mouthfeel), which will be apparent to consumers. However, dietary fibre has a negative image for many consumers due to its perceived association with bloating and gas production. Although there is no objective evidence for this in the case of bread (Weichselbaum, 2012), it is nevertheless likely that at least some consumers will avoid buying white bread marketed as ‘higher fibre’. The alternative approach is to label the fibre content of the new type of white bread in the usual way (i.e. to accurately list the fibre content within the nutritional information but not promote it through marketing). This approach is being increasingly adopted to deliver improved foods to populations with strong cultural food preferences that are resistant to change, including increasing the intake of dietary fibre (Baenziger et al., 2023; Harris et al., 2022).

Finally, it is important to emphasise that increasing the content of fibre in white flour and bread should be part of wider changes in dietary habits required to deliver improved health.

### AUTHOR CONTRIBUTIONS

All authors contributed to the preparation of this manuscript and have read and approved the final text.

## CONFLICT OF INTEREST STATEMENT

The authors report no conflicts of interest to declare that are relevant to the content of this article.

## Supporting information


Table S1.

## Data Availability

Data used to prepare Table 2 and Table S1 are available from the authors. No other new data were used.
